# Supporting the mobilization of health assets among older community dwellers residing in senior-only households in Singapore: a qualitative study

**DOI:** 10.1186/s12877-020-01810-6

**Published:** 2020-10-19

**Authors:** Betsy Seah, Geir Arild Espnes, Emily Neo Kim Ang, Jian Yang Lim, Yanika Kowitlawakul, Wenru Wang

**Affiliations:** 1 Alice Lee Centre for Nursing Studies, Yong Loo Lin School of Medicine, National University of Singapore, Clinical Research Centre, Block MD11, Level 2, 10 Medical Drive, Singapore, 117597 Singapore; 2 Center for Health Promotion Research, Department of Public Health and Nursing, Faculty of Medicine and Health Sciences, Norwegian University of Science and Technology, Postbox 8905, N-7491 Trondheim, Norway

**Keywords:** Elderly, Asset-based approach, Salutogenesis, Community health practice, Sense of coherence, Resource mobilization, Aging assets

## Abstract

**Background:**

Care activities provided by community health practitioners for older adults primarily focused on disease prevention and management. However, healthy longevity can go beyond disease prevention and management and promote greater well-being by tapping into the accrual of resources that older adults possess using the salutogenic approach. This study explored how health resources are used among older adults who are residing in senior-only households to promote and maintain health, with the intent of providing insights into how community health practitioners can support these older adults via asset-based strategies.

**Methods:**

We adopted a descriptive qualitative study design using focus group discussions. Twenty-seven older adults who either lived alone or with their spouses were purposively sampled from an elderly populated residential estate in Singapore. Six focus group discussions, conducted from December 2016 to May 2017, were audio-recorded, transcribed, and analyzed using thematic analysis.

**Results:**

The themes that emerged were ‘tapping on internal self-care repository’, ‘maintaining and preserving informal social support’, and ‘enabling self by using environmental aids’, and an eco-map of aging assets was used to capture an overview of internal and external resources. With the repository of personal strengths, knowledge, and experiences, these older adults were generally resourceful in navigating around their resource-rich environments to cope with everyday life stressors and promote health. However, they were occasionally limited by individual factors that affected their comprehension, access, maintenance, and utilization of resources.

**Conclusion:**

The eco-map of aging assets can be used as an assessment framework by community health practitioners to recognize, consider, and build a repertoire of resources among these older adults. It serves as a gentle reminder to adopt an ecological approach in considering and tapping into older adults’ wide-ranging personal, social, and environmental resources. Community health practitioners can support resource integration as resource facilitators via cognitive, behavioral, and motivational salutogenic pathways to overcome resource mobilization barriers faced by older adults. Such an approach helps older adults to find their internal capabilities and abilities to know who, where, what, and how to seek external resources to identify solutions, creating the intrinsic value to sustain their actions on resource utility.

## Background

Community health practitioners are frontline health professionals with the responsibilities of decreasing risk factor prevalence, reducing acute and chronic disease burden and injury occurrences, as well as being health promotion agents [1]. However, when working with older adults, most care activities centered around early disease detections, risk factor mitigations, and disease management, adopting pathogenic and deficit-based approaches. Healthier longevity goes beyond addressing these disease-focused factors and promotes greater well-being in older adults. Community health practitioners can tap on the accrual of resources that older adults possessed despite the adversities experienced during their late lives [2]. This can be achieved through capacity building via a ‘lifelong process of optimizing opportunities for improving and preserving health and physical, social, and mental wellness, independence, quality of life, and enhancing successful life-course transitions’ [3]. Such an approach of health promotion focuses on health assets and encompasses the idea of adaptability for older adults to adjust physiologically, psychologically, and socially to different phases of their lives [4]. This entails the salutogenic perspective.

The salutogenic approach views health along the ease/dis-ease continuum and focuses on protective factors that facilitate active adaptation to our inevitably stress-rich environments. It comprises the following primary concepts: sense of coherence (SOC) and generalized and specific resistance resources. SOC, which is health-promoting [5], refers to the global perceptual influence of viewing life as comprehensible, manageable, and meaningful [6]. SOC addresses the cognitive, behavioral, and motivational aspects of how one copes with tension caused by stressors [7]. Generalized resistance resources (GRRs) are the ‘physical, bio-chemical, artefactual-material, cognitive, emotional, valuative-attitudinal, interpersonal-relational, and macro-sociocultural’ characteristics ( [6] , p.103) of a person, group, or community that facilitate effective coping of tension caused by stressors [8]. Examples of GGRs can thus be internal (e.g. immunity, wealth, knowledge, happiness, and positivity) or external (e.g. social support and political-cultural stability) characteristics. While GRRs have a wider range of utility, specific resistance resources (SRRs) are activated in specific situations and are context-specific [6], e.g. an elderly who is living alone seeking immediate help from neighbors when in distress. Regardless of the mobilization of GRRs and/or SRRs to overcome stressors, the scope of resistance resources is inevitably linked to the community and ecosystem where people reside in, acknowledging that the social or environmental determinants of health are not within the control of individuals [9]. Coping with stressors intertwines with the interactions of one’s living context and is linked to ecological thinking [7]. An exploration of resistance resources thus involves the consideration of the ecological approach. Ultimately, mobilizing these resources will offer life experiences of consistency, load balance, and participation in valued decision-making, contributing to the development of SOC [6]. Processes of building up an individual’s capacity to use these resources were reported to be more important than merely examining the presence or absence of resources [10].

In the literature, health assets were used synonymously with health resources [11, 12]. Likewise, this study explored resources and health assets as a combined concept. According to a concept analysis, health assets are repertoire of internal and external potentials that an individual possesses, both innate or acquired, that actuate positive health behaviors and optimal health outcomes [11]. Translating the theory of salutogenesis into practice, Morgan and Ziglio proposed to use the theory to examine factors that create health, apply the concept of asset mapping to work on an individual’s capacities, and evaluate community-based approaches using asset-based outcomes [13]. The inventory of health assets may exist at the individual, community, or population level to buffer against stressors encountered by older adults and can be mapped in accordance to older adults’ interactions with their communities and ecosystems [13]. These health assets can subsequently be incorporated in healthy aging interventions that adopt a person-focused or environment-focused approach to address the biopsychosocial health of older community dwellers [14].

However, it is unclear from the literature how community health practitioners can support the use of health assets among older community dwellers. A recent systematic review reported a dearth of studies that examined the health assets of older adults [15]. Hornby-Turner and colleagues reviewed the health assets of older adults by curating personal, social, economic, and environmental factors from quantitative studies that measured associations with composite biopsychosocial health outcomes [16]. They identified self-rated health, life satisfaction, psychological well-being, social networks, contact with family and friends, engagement in leisure and social activities, education, and financial resources. Similarly, most SOC studies conducted on older community dwellers employed quantitative methods to identify health resources by examining relationships between resources, SOC, and health-related outcomes [17–23]. However, knowing how health-promoting resources are mobilized from qualitative findings can be more instrumental than a mere identification of resources for community health practitioners to facilitate asset-based strategies. Factors such as easy accessibility, familiarity to resources, control over resource utility, and the mismatch between older adults’ perceptions and their resource utilities were previously reported to influence resource utility [24]. Nonetheless, the understanding towards resource mobilization among older community dwellers using the salutogenic perspective is limited. As such, a qualitative study using the salutogenic perspective was undertaken to explore how existing health resources are utilized to promote and maintain health in the context of older adults residing in senior-only households in Singapore.

Like in other Asian countries where filial piety is a cherished virtue, family members, particularly children, are traditionally the primary sources of physical, emotional, and instrumental support for older adults. In the recent decade, there has been a notable increase in senior-only households in Singapore [25]. These older adults might have lesser familial resources to cope with late life stressors compared to other older adults who are living with their children. Understanding how this vulnerable group of older adults utilize their existing health resources to promote and maintain health can provide community health practitioners with insights into supporting these older adults in resource mobilization via asset-based strategies.

## Methods

### Design

This study adopted a descriptive qualitative design, drawing principles from naturalistic inquiry to provide a parsimonious but comprehensive description of participants’ perceptions and experiences toward the studied phenomenon [26]. This study is part of a larger study that aimed to operationalize the concepts of SOC and resistance resources to develop a salutogenic health intervention for older adults who are residing in senior-only households in Singapore. While the findings on how these older adults perceived health and aging using the SOC perspective have been reported elsewhere [27], this paper focused on the mobilization of resistance resources and health assets among these older adults.

### Participants

This study was conducted at an elderly-populated residential estate in the western part of Singapore. The residential area was selected for its higher proportion of elderly persons and lower socio-economic status, considering the higher proportion of people residing in one-, two- and three-room apartments. The inclusion criteria were older adults who were (a) aged ≥65 years old, (b) living alone or with their spouses only (≥65 years old), and (c) able to converse in either Mandarin Chinese or English. Older adults with active uncontrolled cognitive and psychiatric conditions were excluded. Older adults with different combinations of living arrangement and gender characteristics (e.g. elderly man living alone or elderly women living with spouse) were purposively selected.

### Procedure and data collection

Focus group discussions (FGDs) were employed as a data collection strategy to tap into the synergistic effect of interactive discussions among participants with similar backgrounds. This identified shared prominent issues and generated wide-ranging perspectives [28]. Considering the wealth of health experiences that these older adults might share, their loquacity, and the ease of personal sharing in small-group discussions, smaller focus groups were employed.

Several recruitment strategies were adopted to capture the views of older adults with different levels of social participation. This included approaching older adults during a senior event held at a community center, common public spaces, and through door-to-door knocking. Eligible interested older adults were contacted via telephone calls and subsequently grouped according to their preferred spoken languages. Four FGDs in Mandarin Chinese and two FGDs in English language were conducted before data saturation was met.

All FGDs were held at a community center and recorded audially. The average duration of each FGD was 114 min, ranging from 89 to 151 min. All FGDs were moderated by the same bilingual researcher, and she was accompanied by a note-taker. To generate effective discussions and obtain rich data, efforts were taken to build rapport with the participants, focus on the research aims, establish ground rules, promote group cohesion, manage group dynamics, and pace the discussions [28].

The semi-structured interview guide was developed based on a literature review [16, 17, 19–21] in accordance to address the SOC and GRRs of older adults [9]. Interview questions were phrased to illuminate the participants’ health and aging experiences and reveal the resources needed or used to cope with late life stressors through health-promoting actions [27]. Both recruitment and data collection occurred from December 2016 to May 2017.

### Data analysis

The audio-records were transcribed verbatim in their original languages either by the moderator or another bilingual research member. Braun and Clarke’s thematic analysis was used as it comprised a two-stage integrative process of reviewing themes against its codes and the entire data set to identify patterns within and across the entire data [29]. Firstly, the two researchers immersed themselves into the data by checking the transcripts against the original audio-recordings twice, followed by reading and re-reading each transcript line-by-line. Secondly, they independently generated initial codes by looking out for segments of textual data that portrayed ideas related to the research question. Thirdly, the codes were sorted, organized, and combined into broader levels of sub-themes via the aging asset. These sub-themes collapsed into themes such that the aging assets were organized at the individual, community and societal/population level (refer to the supplementary table). The fourth step involved a two-stage review process at the extracted data level and the entire data set level to re-organize the collapsed codes and potential themes until they fitted together. Fifthly, each theme was refined and defined in English before it was reported using vivid extracted quotes and examples. This entire process was iterative. The two researchers had extensive discussions and inquiry exchanges that tested their developing insights and probed deeper explorations of questions. Data were analyzed in its original verbatim language through independent coding before a consensus was achieved on the codes and themes in English. This minimized misinterpretation risks and prevented losses of the participants’ intended meanings within the sociocultural context. Discussions were held with the research supervisor to resolve differences and clarify the categorization of codes.

Although the salutogenic theory was used to develop the interview guide and discuss the findings in this study, it was not used to analyze the data deductively. Instead, data were analyzed inductively with the consideration of ecological thinking to identify and explore SRRs or health assets that mattered to the studied community and its living context. Unlike GRRs, there is no clear theoretical scope for SRRs.

### Ethics

Ethics approval was obtained from the university’s institutional review board. Prior to the conduct of the FGDs, written and verbal information concerning the study’s aims and procedures was provided. Time was allocated to address queries before obtaining written consents. To ensure the participants’ confidentiality, pseudonyms were used.

## Results

A total of 27 older adults participated in this study. Their ages ranged from 66 to 79 years old. Most of them were retirees. Among them, 13 lived alone and 15 were female. Although all of them resided in public housing high-rise apartments, their socio-economic statuses differed. Their residences ranged from rented studio apartments to self-purchased five-room apartments. The physical health profiles of the participants included having no chronic diseases, at least one chronic disease (e.g. hypertension, diabetes mellitus, or hyperlipidemia), and multi-morbidities involving significant major organ conditions (e.g. heart, lung, or brain). Table 1 summarizes the participants’ demographic characteristics.

**Table 1:** Participants’ demographic details

Characteristic	Total (***n*** = 27)
**Age** (range in years)	73.07 ± 3.80 (66–79)
**Gender**	
Female	15
Male	12
**Living arrangement**	
Live alone	13
Live with spouse	14
**Marital status**	
Married	14
Widow	5
Divorced/separated	4
Single	4
**Ethnicity**	
Chinese	23
Malay	3
Indian	1
**Ownership of residence**	
Self-owned	23
Rental	4
**Employment status**	
Full-time	2
Part-time	4
Unemployed	2
Retiree	19
**Presence of chronic diseases**	
Yes	24
No	3

**Fig. 1:**
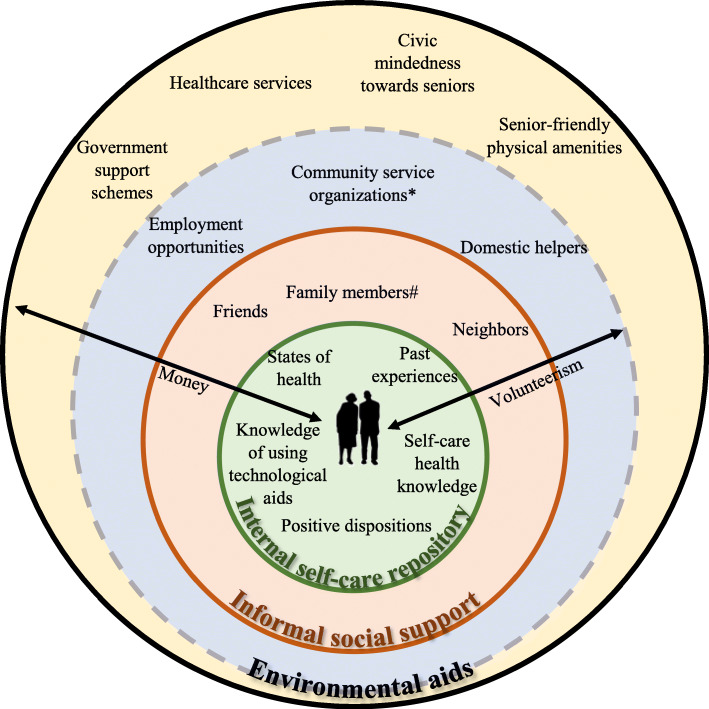
Eco-map of aging assets. #spouse, children, and relatives *community centers, senior activity centers, social service organizations, and places of worship

The emerging themes were ‘tapping on internal self-care repository’, ‘maintaining and preserving informal social support’, and ‘enabling self by using environmental aids’. These encapsulated the description of existing key individual, social, and environmental aging assets (Fig. 1). The participants mobilized these health assets to actuate health promoting strategies to cope with late life stressors. The findings uncovered the older adults’ barriers in resource mobilization related to accessibility, maintenance, and know-how utility.

### Tapping on internal self-care repository

Each participant had his or her unique repository of internal resources, such as his or her state of health, positive psychological disposition, self-care health knowledge, past experiences, and knowledge of using technological aids. The participants drew upon their resources to provide self-care and promote their physical, psychological, and social health.

#### States of health

Having good health gave the participants functional independence. It was an asset that all of them desired and valued the most. However, one’s state of health could affect functional abilities that actuated or hindered the pursuit of what one perceived as meaningful.

*“We want good health, with our hands and legs functioning, so that life will be easier. We can go anywhere we like, do the things we want.”* (Chew See, 73).

#### Positive dispositions

Having positive dispositions helped the participants to cope psychologically with stressors faced during their late lives. It also facilitated the sustainability of health actions. This included positive attributes such as contentment, optimism, broad-mindedness, perseverance, and self-discipline. Despite having multiple co-morbidities, some participants were contented with their current health states and living circumstances. They appreciated the positive aspects of what they had, which brought them happiness. Ying Ying’s optimistic outlook made her carefree and gave her joy in her day-to-day activities. Being able to lighten-up, see things from a wider perspective, and let go of clinging views were commonly mentioned. Meng Hoe said, *“Must let go. Do not dwell on it. One must adjust to one’s own self. If the mountains do not move, you move.”*

The participants tapped into positive traits such as self-discipline and perseverance to keep themselves going and maintain health actions. While four participants identified that self-discipline put health actions into daily routines, others shared how their perseverance led them to achieve better health statuses. Ah Chye attributed his full recovery from a heart bypass surgery to his persistent efforts in daily morning walks.

#### Self-care health knowledge and life experiences

The participants tapped into their health knowledge and life experiences that were accumulated from years of interactions with their environments to take on positive self-care actions. When Meng Hoe was working as a hawker assistant, he had to prepare chili sauce and stir-fry dishes with leftover dark cooking oil. This made him acutely aware of undesirable food preparation methods by food vendors and activated him to consume homecooked food. Likewise, Wah Lee used what she had learnt during her school days to guide her current daily exercises.

The participants retrieved and gathered self-care health information from multiple sources, such as word-of-mouth, library books, newspapers, radio broadcasts, television shows, and healthcare professionals. They had to discern the information that they received.

*“I listen strictly to doctors … Even if your friends were to give you comments, try and weigh whether to follow them … You must think, take a step back … Evaluate whether is it okay to go ahead or not.” (Peh Chin, 75).*

Apart from consulting doctors only when ill, the participants hoped that they could approach health professionals for advice whenever in doubt. Content from various information sources might be unreliable or misinterpreted by the participants. There were several occasions during the FGDs when a few participants shared misleading health tips with their peers.

*Wah Lee: “When I was about 60kg, my doctor asked me to lose weight. I told him that I swim. He said, ‘Don’t even drink coffee after swimming. You can gain weight from drinking it. When you are obese, (it) put pressure to your legs (knees).’”*

*Eng Hwa: “Cannot drink coffee?”*

*Wah Lee: “Not that you cannot drink. It makes one put on weight.” (FGD 1).*

#### Knowledge of using technological aids

Only some participants described how they used technological devices to promote psychosocial and cognitive health. Examples included smartphones and tablets that they used to communicate with family members and friends, play online game applications for leisure, and stimulate cognitive thinking. Despite the existing information technology (IT) courses provided for seniors as part of the Singapore government’s initiative to be a smart nation, the participants hoped for additional support whenever they required personal technical assistance.

*“Perhaps, there is a particular place where people like me … can turn to to ask certain questions about computers, smartphones … not the SkillsFuture fund*^1^*whereby you attend three hours of course … But specific questions … one or two questions … And the best is to be personal, one to one … rather than over the phone.” (Keng Gim, 77).*

### Maintaining and preserving informal social support

The participants reported the need to stay connected with people, including their children, spouses, relatives, friends, and neighbors. As most participants treasured their relationships, they made attempts to establish connections and maintain their dwindling social networks. However, a few struggled to preserve them.

#### Family members

Despite the separateness in place of residence, children, siblings, and other relatives were cherished sources of emotional and instrumental support. When such people extended occasional greetings or visited the participants, it brought feelings of joy, pride, comfort, and self-importance, which boosted psychological health. Hai Wee added that he could boast to his friends that his children were filial*.* Apart from receiving monthly allowances from their children, most participants still relied on family members for assistance in times of sickness or situational vulnerabilities. A few seniors reflected that while the legislation^2^ mandated children to provide care for their parents, representations and portrayals of affection and care differed between families. Some participants aired concerns about filial piety being an important yet eroding attribute. Almost all participants kept in contact with their children and siblings, maintaining kinship in one way or another. However, the depths of interactions varied as the frequency of tele-conversations and face-to-face meetups with family members ranged from a few times weekly to a few times annually.

Participants who were living with their spouses demonstrated strong interdependent relationships. Several affirmed love and respect for their spouses and would take care of their spouses despite any odds faced. Although caring for her sick husband was tough, Ting Lay said that she would take care of him if she had the means to take care of him. Despite quarrels, most married participants maintained their relationships with spouses by giving in and embracing each other. They were each other’s resource, providing mutual emotional support and companionship. However, such resources diminished when one of them passed on or required dependent care.

*“I have been with my wife for 62 years marriage. She is my backbone … I treasure spousal love the most … until death does us apart. Love one another, understand one another, respect, sickness … My wife plays a very big part in my life.”* (Roslan, 78).

#### Friends

To most participants, friends were vital in providing companionship and bringing moments of jollity through activities or times spent together. These moments with friends made the participants feel happier, forget their worries, and feel less lonely. With fewer family interactions, friends were particularly important to those living alone. Otherwise, such participants felt that they had nothing to do daily. Having friends in close proximities allowed the participants to rely on them in times of need. Recognizing its importance, the participants selectively made friends with people of similar interests and who they could get along with.

*“I try to mix [around] more with people. And I just handed him (another participant) my phone number, since he is also solo. Make friends, you know. There could be something common between us.”* (Keng Gim, 77).

It was observed during the FGDs that when the participants exchanged experiences and knowledge with each other, they gained new information from their peers. Peers were resources for not only emotional sustenance but also tangible and informational support.

#### Neighbors

Although the participants recognized that they could approach their neighbors for assistance when they were in need of situational tangible support, neighbors were untapped resources. Only several participants trusted and built relationships with their neighbors. They could rely on their neighbors when they were in need of help for day-to-day household chores or urgent medical attention, e.g. falling at home.

Hai Wee: *“Neighbors are important because they are the closest to us. If [we] need anything, [we] can help each other, talk to and ask each other out for activities … They may look out for you … Now, good neighbors are hard to come by.”*

Chew See: *“Now, don’t have such neighbors... It’s more that you mind your own business, I mind my own business.”*

Keng Siew: *“Yah. We usually close our doors when we return home.”* (FGD 5).

A third of them shared their challenges of establishing neighborliness, which included language barriers and opportunities to bond.

*“My neighboring units are rented to Indians. How to communicate with them? Moreover, the (Chinese-speaking) younger neighbors will go out to work. [They are] seldom at home.”* (Di Meng, 70).

### Enabling self by using environmental aids

While some participants were savvy in exploring and reaching out to community resources and formal support services, others were less resourceful. These environmental aids, when mobilized appropriately, enabled and empowered them to cultivate health-promoting actions, spur positive emotions, or promote self-care.

#### Money

Money, also identified as ‘vitamin M’ by the participants, was generally the second imperative resource after health. It was perceived as a preceding resource to fulfill basic demands of everyday living, such as food, transport, healthcare, and individual desired pursuits. Although many raised concerns about having financial security to subsist the remaining years of their lives, only three participants emphasized the importance of prior financial planning for old age.

Money is both an internal and external resource. Most participants drew sources from personal savings, Central Provident Fund retirement withdrawals,^3^ allowances provided by their children, housing rental incomes, and/or existing employment salaries (if any). However, for participants who had difficulties in making ends meet, ‘vitamin M’ was primarily an external resource. They would deplete personal and intermediate family members’ savings before they sought help from social workers, social service organizations, and government social assistance schemes.^4^ Having insufficient personal wealth or excessively dwelling over finances were troubling and stressful and were thought to bring down the participants’ health.

#### Domestic helpers as potential resources

Although the participants lived by themselves, they were aware of and suggested hiring foreign domestic helpers to relieve daily workloads. These helpers were potential ‘available’ resources that older adults could employ when they needed additional pairs of hands to perform household chores or care for themselves or their spouses. Lian Lee who ambulated with a mobility scooter lamented that inadequate finances limited her from hiring a domestic helper. Contrastingly, Ting Lay held on to her pride and autonomy and refused to accept external hired aids by her son despite struggling to cope with day-to-day care for herself and her husband who had dementia. She did not like having too many people in her house as it made things too complex for her and preferred to have freedom at home.

#### Employment opportunities

To both working and non-working participants, holding a job was healthful. They mentioned that employment not only engaged them temporally, mentally, physically, and economically but also contributed to their self-worth.

*“He [another participant] has that qualification … experience. Surely, there is some way that he can contribute to the economy. And for his health also! It’s related. Stress. He misses his wife. Would this be a distraction away from his thoughts of his [late] wife? It helps a lot … Furthermore, he still has an economic life! He is not zero.” (Keng Gim, 77, responding to a 75-year-old male participant’s sharing on unsuccessful job interviews).*

Depending on individuals’ preferences and circumstances, several participants desired to be employed. They attempted to look for jobs, which was in vain. Some reasons shared were disapproval from their children, difficulties in gaining access to preferred jobs due to age, disinterest in certain jobs, and mismatched physical capabilities.

#### Community service organizations and volunteerism

Apart from carrying out home activities to stay occupied, the participants had to locate, distinct, and enroll in activities and services that were provided by the existing array of community service organizations. Such community resources comprised the neighborhood’s community center, senior activity centers (SAC), social service organizations (SSO), and places of worship (e.g. churches, temples, and mosques). Some of which provided senior-centric programs and services. Among participants who were aware of these community resources, some showed misunderstandings or a lack of understanding toward the purposes, entries, or types of services and activities that organizations provided. Wah Lee shared her preconception and discontentment towards SSO-supported SACs that catered to needy seniors only and discounted activities at other senior centers that were open to all seniors.

Only some participants appreciated these centers as communal areas that were designated for seniors to convene, mingle, and participate activities to occupy themselves and stay healthy. One participant broached the subject on the lack of participation and interest among senior residents to utilize these areas. Others expressed their individual priorities, diverse preferences for activities, and low confidence for activity participation in these areas, revealing potential mismatches of seniors’ needs and the resources that were provided. A few participants suggested building more senior centers with a greater variety of programs and an active and tighter management to facilitate the regulation and running of such centers.

*Hai Wee: “They always encourage seniors to go. Get your friends or neighbors to go together. They encourage us to come down for activities, not to stay at home the whole day and watch television as it is not good for our health. But efforts are futile. [It] seems like no one wants to go.”*

*Lian Lee: “Some people don’t like to participate in activities. They prefer solitude.”*

*Hai Wee: “There are massage chairs, newspapers to read, rummy-o (board game) … You do not need to pay for the (massage) equipment. Sometimes, there are talks. On Tuesdays, there is a dance class and you don’t have to pay to attend it. But (some) people still don’t want to go, even those who live in the same block above the center.”*

*Keng Siew: “But it’s difficult. Some seniors’ legs are not good. How can they dance?”*

*Hai Wee: “If they cannot dance, they can sing karaoke.”*

*Kim Meng: “I won’t participate in these activities. I don’t like them. Different people have different interests. I prefer to walk around on my own and spend time alone … They are afraid that seniors keep staying at home.”*

*Ting Lay: “It’s better to participate … If you don’t participate, you can get dementia easily … It’s better to go. But if you don’t have time, it can’t be helped. I will go if I have the time.” (FGD 5).*

Some participants also benefited from these community resources through volunteerism, either by being active volunteers or recipients of volunteering and social services.

*“Seniors living alone need someone to visit them weekly, or else [they] will grow worms (become useless and expire soon). So, we do house visits for them and it is an activity for us … Now, [I] still have the energy to do so...” (Nee Mui, 66).*

*“I am on meals-on-wheels, by [name of SSO] … When I need to see a doctor, there is a van arranged to transport me there. I received a [mobility] scooter too … There are volunteers and three social workers taking care of me.” (Ah Leong, 78).*

#### Government support schemes

Some participants perceived government support schemes as resources, placing their faith and dependence on the government to provide them with better healthcare and living environments. They were generally appreciative of the existing support schemes and public services put in place. Some described how certain support schemes incentivized them to take up desirable behaviors, such as learning new skills and knowledge using SkillsFuture Credits and using ActiveSG credits to exercise. However, mismatched intents on the use of such schemes were observed. Few participants verbalized that upon depletions of their free credits, they discontinued their learning activities. Others mentioned other government support schemes that provided subsidies and relief for seniors to receive affordable healthcare.

*“Now, if we want to keep our minds active, we can use the $500 [SkillsFuture] credit given by the government … You just need to register for the class that you like … I have used … to attend courses on how to do business, how to engage others in conversations, and English classes too.” (Ching Ai, 75).*

#### Healthcare services

When ill, the participants entrusted their health to health service providers to manage their diseases. They knew that they could access these resources by consulting either public or private medical providers to diagnose their conditions, receive treatments, or seek second opinions. Generally, the participants placed high reliance and regard for healthcare professionals’ advice and treatment plans to manage their health. Only one participant disbelieved her doctors and was non-compliant to her medications.

#### Senior-friendly physical amenities

Some participants highlighted the importance of senior-friendly physical amenities within and outside home environments to promote personal safety and encourage health-promoting behaviors. They took home safety and fall precautionary measures such as installing grab bars, maintaining household hygiene, and minimizing hoarding behaviors. Others appreciated and used public infrastructures within their neighborhoods. These included the extended countdown timer at pedestrian crossings to facilitate mobility, parks offering green spaces and fresh air, and fitness corners for seniors to exercise, gather with friends, and relax.

*“Our living environments need more exercise facilities (physical therapy equipment) that are suitable for seniors. It will be a concern if [there is a] lack of such (facility)... to keep fit.” (Meng Hoe, 75).*

#### Civic mindedness towards seniors

Lastly, the participants added that they valued positive senior-friendly social environments. Encountering strangers who respected them as seniors and embraced their physical health needs was heart-warming. It made them feel welcomed and acknowledged.

*“I ordered my coffee and left my last capsule of medicine packet on the table … and here comes … I thought that she was a table cleaner. And she said, ‘Uncle, you are taking a certain medicine. Can I offer you some water?’ That touched me … All these years that I have visited a* kopitiam *(coffee shop), I have never encountered such a thoughtful pleasant lady … Now, look at my age. Never have I experienced such an encounter... I felt very pleased. And when a person feels pleased, it is also good for [his or her] health. So, this should be spread.” (Keng Gim, 77).*

## Discussion

This study explored how existing health resources were utilized among older adults residing in senior households to promote and maintain health, with the aim of understanding how community health practitioners can support them in resource mobilization to live healthier lives. Using concepts on resistance resources and ecological thinking arising from the salutogenic theory, the eco-map of aging assets emerged. It captured an overview of intrinsic and extrinsic resources, including contemporary trends such as having the knowledge to use technological aids, hiring domestic helpers to attend care needs, acts of seniors volunteering, and the appreciation of civic mindedness towards seniors. While most of the emerged health assets were not new in the literature, the use of the qualitative approach, compared to quantitative measures, allowed the identification of health resources that mattered to these older adults and unraveled the challenges and issues faced during resource mobilization by this specific community. This section discusses the challenges and issues arising from the mobilization of personal, social, and environmental resources and how community health practitioners can employ asset-based strategies to support them. Using the eco-map of aging assets as an assessment framework, community health practitioners can take on the role of resource facilitators to assist these older adults in understanding, accessing, maintaining, and utilizing their aging assets.

### Personal repository

Apart from having good health [27], having a positive disposition was an asset to healthy aging. It took forms in multiple good individual traits, such as being optimistic and contented, exhibited either through the participants’ narrations of personal experiences or direct attributions [30, 31]. The participants harnessed their strengths to hone positive orientations in coping with late life challenges and taking charge of their health psychologically and behaviorally. Consistent with past studies, positive outlooks toward changes in their own health and aging experiences gave older adults a sense of willpower and control over their health and lives [24, 30, 32]. Recognition and acknowledgement of older adults’ personal strengths by community health practitioners thus become important in revealing older adults’ individual traits, principles and attitudes each of them prized. Attitudes toward life impacted older adults’ decision-making of how they cared for themselves [33]. To better tap into these health assets, community health practitioners can facilitate older adults to be cognizant of and apply their personal strengths, values, and principles in coping with late life stressors as well as in self-care.

Older adults’ self-care knowledge was a product of accumulated experiences and exposure to information, which they perceived to be beneficial in enhancing their health and well-being. It is an asset related to health literacy and requires one to access, understand, appraise, and apply health information and services to promote and maintain health [34]. Consistent with a past study conducted in Singapore [35], older adults obtained health information from multiple sources and had to determine such information’s trustworthiness. In most situations, older adults reached out to personal social networks for health information, and less frequently via online sources [36]. Although abundant health information is available online in this digital age, most study participants received little education and were not proficient in English or savvy in navigating online platforms. Being recipients of information from multiple sources, they identified healthcare professionals from formal channels as credible sources, e.g. healthcare institutes and health education programs [37]. Thus, they hoped for more accessible touchpoints with community health practitioners to address their queries related to self-care and health-promoting actions. Community health practitioners thus need to play more visible and active roles in promoting health literacy and be reachable to older adults on community platforms to provide health information assistance.

This qualitative study captured how some participants leveraged on technological aids in their everyday lives, e.g. smartphone and tablet applications for leisure activity participation, maintaining relationships with loved ones, and personal development. At times, such health assets became problematic when these participants required brief quick assistance in troubleshooting technical problems. In contrast with some older adults living alone who lacked basic communication technology and skills a decade ago in Singapore [37], older adults today have advanced in technological device usage and are given opportunities to learn and upgrade their IT skills and knowledge. Acknowledging that such learning resources are available and that older adults engage or disengage in technological platforms for various motivations [38, 39], community health practitioners can play supportive roles in encouraging or assisting interested older adults to hone their technological-related skills and knowledge as part of the health promotion efforts to engage in valued activities.

### Informal social support

Consistent with past studies, the participants’ social resources encompassed family members, spouses, friends, neighbors, and the presence of social and leisure activities [24, 30, 31]. As social connectedness is a valued aspect of health among older adults [40], the participants from this study longed for and sought social interactions with others. Apart from fulfilling emotional and social needs, the participants reported that they relied on their social assets for tangible assistance and information support. However, when the participants’ expectations toward their social environments were not met, relationships and interactions with these social assets could become stressors [24] and impact their psychosocial health. Filial piety, perceived as an important virtue in care provision for elderly parents [41], was thus questioned when the strengths of relationships and interactions with their children fell short.

Soon et al. [42] stated that interdependence and independence in marital relationships among older adults living with their spouses only were valued and intertwined. As such, reciprocal support and mutual caring between late life couples altered from asset to stressor when one passed on or when fulfilling caregiving roles for an ill-dependent spouse [43]. Quality of marital relationship, which is an asset, can also be affected when one from the dyad falls ill [44]. Perhaps for late life couples, promoting self-independence and learning to reduce reliance on their partners for daily living or household-related matters will partially ease their transitions and adaptations as such social assets gradually diminish with their spouses’ ill-health or death.

Friends are also an asset, particularly to older adults living alone [45]. Engagement with friends through activity participation contribute to emotional health and can encourage positive health behaviors [30, 31, 46]. The findings also reflected that friendships offered solidarity in aging, whereby friends helped each other when they were in need and shared useful information within the community [30, 31]. While this study observed that older adults made friends through participating in common activities, Chong et al. [47] added that friendships were forged when they interacted at both community-based centers and informal public spaces within the neighborhood. With the dwindling of social networks overtime [27], building and maintaining new friendships among older adults become crucial. While friendship is an interpersonal interaction, the creation of opportunities to establish and maintain friendship can be propelled by activities organized by community health practitioners, services provided by community organizations, and shared community spaces.

The findings revealed that neighbors were untapped and underutilized assets despite their significance for proximal immediate help in times of need. Although the participants resided in the proximities of their neighbors in public housing high-rise apartments, a number of them withdrew themselves and failed to establish neighborly relationships. Only a few reported that they could rely on neighbors when in distress. Unlike a study conducted in the Netherlands [24], older adults residing in rural areas could get assistance from neighbors in spite of minimal contact with them. Our findings also differed from previous local studies [45, 48]. They reported that older adults living in studio apartments and rental public housing units had prior mutual arrangements with neighbors to keep a lookout for each other. Although the present study observed that participants who could rely on their neighbors resided in rental apartments and those with minimal contact with their neighbors lived in self-owned three-room to five-room public housing apartments, it is unclear whether socioeconomic status plays a role in neighborly relations and exchanges in the community. Future studies and initiatives can follow up on these available but underutilized assets.

In view of older adults residing in senior-only households who face dwindling social connections [26, 48], community health practitioners can focus on building new and strengthening existing social assets. These include organizing activities and creating spaces for older adults to engage in meaningful exchanges and establish new friendships, as well as encouraging social interactions with family members, existing friends, and neighbors to deepen or maintain relationships.

### Environmental aids

Having financial security is an important asset as it empowers older adults and makes them feel safe [49]. Unlike developed countries with welfare states, citizens in Singapore are encouraged to be self-reliant through asset accumulation to care for themselves and elder family members. Thus, unexpected occurrences of late life events, such as illnesses that incur high medical expenses, can jeopardize older adults’ future financial subsistence and security. Consistent with past local studies, financial security has been a concern at late life [37, 50]. Compared to objective financial stability, perceived financial stability had a greater influence on psychological health among older Singaporeans [50]. Moreover, having financial assets in late life contributed to life satisfaction [51]. This underlined the importance of personal financial planning prior to and during old age, not only for self-reliance but also as part of self-care and health promotion efforts at large. It gives older adults greater control and, thus, the financial abilities to pursue desired meaningful activities. However, this will be challenging for individuals who already face financial difficulties and have to rely on social services for day-to-day living subsistence.

Perceived as potential assets by the participants, employing live-in foreign domestic helpers may reduce daily household workloads and caregiving responsibilities for older adults and/or their ill-dependent spouses. This was evidenced by local studies that reported better caregiving outcomes [52] and provisions of socio-emotional support for older adults who were less mobile [53]. However, having an external ‘pair of hands’ living and providing care in a household is not without its complexities [53]. The present study reported some of the participants’ deliberations, personal preferences, and financial difficulties in hiring live-in domestic helpers. While existing local schemes such as levy concessions and home caregiving grants are available to support older adults with these potential assets, community health practitioners can also offer alternative care options to cater to their needs [54].

The participants spoke positively of late life employment being a meaningful activity that benefits overall wellness. This corresponded with evidence on protective effects of meaningful employment on older adults’ functional, physical, social, and cognitive health [55–58]. Community health practitioners can encourage older adults to take up jobs, regardless of full-time or part-time, as a form of health promotion activity rather than for the purpose of earning for a living. However, some participants shared their challenges in searching for and accessing employment opportunities. While there are existing support schemes and services related to job redesign, job matching, and job training for seniors in Singapore [59], interested older jobseekers need to understand how they can contribute with their skills and experiences in dynamic workplaces, learn about the function and purpose of these schemes, and, lastly, enroll in them. Future research can delve into understanding how late life employment can be made attractive as a health promotion activity for seniors to stay engaged and how industrial partners can support this initiative.

Community service organizations are valuable platforms in providing older adults with opportunities to mingle, participate in different types of leisure activities, and volunteer their services. These boosted older adults’ health. However, some participants could not navigate and utilize these services due to their lack of awareness or preconceived understanding toward service provisions. This study uncovered some reasons for the underutilization of community-based centers, such as the lack of time and interest in activities [47] as well as poor activity engagement and empowerment experienced by users of SAC [48]. The findings suggested the need for greater communication efforts and outreach to improve the accessibilities of existing community-based services. With a better understanding of older adults’ activity preferences and needs, activities can be reoriented to improve the utility, experience, and inclusiveness of community-based senior services.

Depending on the nature and type of government support initiatives, these played supportive roles in making healthcare affordable and facilitating older adults to engage in healthy behaviors, e.g. performing physical activity, learning new skills, and working. In 2015, the Singapore government launched an ‘Action Plan for Successful Aging’, comprising more than 70 initiatives across various diverse areas such as health and wellness, learning, employment, public spaces, respect and social inclusion, retirement adequacy and healthcare, and aged care [60]. As these initiatives were implemented over a few years, this study captured how the participants utilized some of these initiatives. It was observed that participants who stayed abreast of these support-schemes perceived these as resources that they leveraged on. On the contrary, there were participants who were not cognizant of these assets despite the various forms of communicative efforts to reach out to older adults. Perceptions on information received on the support schemes varied and this influenced older adults’ intentions and motivations to use the resources, e.g. they would discontinue upgrading themselves through courses upon depletions of the incentives provided by the support scheme. Mismatches on how information on resources were presented and how older adults perceived them were also previously reported to influence older adults’ intentions to use resources [24].

### Community health practitioners as resource facilitators

The findings shed light on how community health practitioners can adopt asset-based approaches in supporting older adults who are residing in senior-only households in resource mobilization to live healthier lives. The preceding discussion reflected varying levels of resourcefulness among the older adults, surfacing barriers related to the comprehension, access, maintenance, and utilization of the identified health assets. At times, these were limited by individual factors, e.g. negative dispositions toward late life [27], poor health knowledge, and inaccurate perceived usages of aging assets, to navigate around external resources. The personal, social, and environmental resources in the eco-map of aging assets converged and integrated at the individual level to cope with everyday life stressors and facilitate health-promoting outcomes. Community health practitioners can thus support in resource integration as resource facilitators to overcome resource mobilization barriers faced by older community dwellers. Particularly, community health practitioners can pay attention to older adults who are less resource savvy in coping with daily life challenges, maintaining self-care, and adopting positive health behaviors.

Salutogenic health development includes promoting an individual’s capacity in resource mobilization [10], and processes of resources mobilization can contribute to the development of SOC via its cognitive, behavioral, and motivational component characteristics [5]. Integrating these theoretical concepts with this study’s findings, capacity development in resource mobilization among older adults can be facilitated in practice according to the SOC’s three cognitive, behavioral, and motivational characteristic components to overcome resource mobilization barriers related to comprehension, access, maintenance, and utilization. To address the cognitive component of SOC, community health practitioners can facilitate older adults to be aware of, comprehend, and discern the types of available resource. To address the behavioral component, community health practitioners can support older adults in adopting the know-how knowledge of initiating and maintaining utilities of specific resources. Lastly, to address the motivational component, community health practitioners can facilitate older adults to make sense of the intended purpose of using a specific resource to achieve desired meaningful activities and cope with everyday life stressors. For example, a minimally educated older adult who is interested in learning how to use social media platforms to interact with his or her loved ones but is unsure how to do so can be guided by a community health practitioner by introducing available courses in the vicinity and providing some basic technical knowledge (cognitive), initiating online interactions with him or her through these communication platforms (behavioral), and commending his or her efforts in learning to maintain contact with loved ones (motivational). This asset-based strategy thus facilitates the sustainability of adaptable health orientation through comprehension, actions, and intrinsic values to continue resource utility. Such an approach contrasts with traditional health promotion that focus on the risk minimizations, early detections, and managements of diseases and shifts the attention to more wellness-oriented and person-centered health goals, e.g. relationship building. To embrace this salutogenic way of care provision, the roles of community health practitioners will thus need to be altered, expanded, or created to empower older adults’ personal capacities to mobilize surrounding resources in the community.

The germinal eco-map of aging assets can be used as an assessment framework by community health practitioners to recognize, consider, and build repertoire of resources within and among older community dwellers residing in senior-households to cultivate strength-based approaches in health management [13]. This framework demonstrated the multiplex dynamic ecological interactions between older community dwellers and their resource-rich living environments. Depending on the situations and values that older adults place on their aging assets, these can be either resources or stressors [6]. For example, spousal support can be a resource or cause of stressor when absent or inaccessible (e.g. when a spouse passes away or becomes ill). In other words, taking on the role of a resource facilitator requires a community health practitioner to have knowledge about older adults’ internal and external resources before being able to provide useful personalized support in resource navigation. The use of such asset-based strategies also involves identifying older adults’ personal strengths, understanding their perspectives and health goals [61], being familiar with community resources and social and health policy schemes [62], and working collaboratively with older adults to meet their stressor demands and pursue meaningful endeavors.

### Limitations

While the eco-map of aging assets provides an overview of the multiplex interaction between an older person and his or her resource-rich environment, the health assets emerged from this study might not be exhaustive. Although older adults residing in senior-only households can comprise individuals staying with elderly relatives or other unrelated elderly, this study did not include this subgroup population. Their daily living experiences and interactions with social resources may differ from those living alone or with spouses only. As the interviews were conducted at a community center, older adults who were home-bound or could not get out of their houses independently were inadvertently excluded. Nonetheless, this qualitative study included older adults with multiple co-morbidities and those who used mobility aids to get around [27]. Additionally, this study was conducted in a residential estate that consisted of families with relatively lower socioeconomic statuses in Singapore. The findings emerged may be cultural and community specific. They may not be generalizable to other elderly populations.

## Conclusion

In summary, older adults residing in senior-only households were generally resourceful in navigating around their resource-rich environments to cope with everyday life stressors and promote health. However, they were occasionally limited by individual factors that affected their comprehension, access, maintenance, and utilization of aging assets. As such, community health practitioners can support them as resource facilitators. The eco-map of aging assets from this study can be used as an assessment framework by community health practitioners to recognize, consider, and build repertoire of resources within and among these older adults to cultivate asset-based approaches in health management. It serves as a gentle reminder to community health practitioners to adopt an ecological approach in considering and tapping into older adults’ wide-ranging personal, social, and environmental resources. This helps community health practitioners to use an asset-based approach to find older adults’ internal capabilities and abilities to know who, where, what, and how to seek externally to find solutions. More significantly, this study advances knowledge on the operationalization of resource mobilization via cognitive, behavioral, and motivational pathways to address the challenges faced by these older adults using the salutogenic perspective.

## Supplementary information

**Additional file 1.**

## Data Availability

The generated and analyzed data set will not be made available to ensure the participants’ confidentiality, as indicated in the information provided to them during consent taking.

## Abbreviations

SOC: Sense of coherence
GRR: Generalized resistance resources
SRR: Specific resistance resources
FGD: Focus group discussion
IT: Information technology
SAC: Senior activity center
SSO: Social service organization

## Footnotes

1 The SkillsFuture scheme, which is part of a nation-wide movement, provides individuals with an opening credit of SGD$500 to encourage the ownership of skill development and promote life-long learning. Training programmes under this scheme include IT-related courses for citizens.

2 The Maintenance of Parents Act seeks to protect Singapore residents who are aged ≥60 years old and unable to maintain themselves sufficiently by allowing them to claim maintenance from their children who are capable of supporting them.

3 The Central Provident Fund is a compulsory social security saving scheme that supports retirement security, home ownership, health care, education, insurances, and investments for working Singapore citizens and permanent residents. It is an employment-based scheme that includes mandated savings contributions from both employees and employers.

4 Singapore’s social safety net is based on the principles of self-reliance, with family as the first line of support, and the ‘Many Helping Hands’ (MHH) approach. The MHH approach is a community-based framework that allows social service organizations, donors, volunteers, grant-makers, and government bodies to provide social assistance to poor, vulnerable, and disadvantaged individuals.
