# Supplementary material for: Supporting the mobilization of health assets among older community dwellers residing in senior-only households in Singapore: a qualitative study
Source: BMC Geriatr. 2020 Oct 19;20:411. doi: 10.1186/s12877-020-01810-6 (PMC7574307; doi:10.1186/s12877-020-01810-6)
Supplement: Supplementary file 1 — Additional file 1. [file 12877_2020_1810_MOESM1_ESM.docx]

Supplementary table to describe the data analysis process

| Selected examples of Quotes | Codes | Sub-themes (aging asset) | Theme |
| --- | --- | --- | --- |
| *‘We want good health, with our hands and legs functioning so that life will be easier. We can go anywhere we like, do the things we want’* (Chew See, 73)  *‘Even if I can live till 100 years old, it does not mean anything. If you cannot walk, what can you do? Isn’t it more suffering?’* (Ah Soon, 75) | - Health is important - Wants to be healthy - To be physically functional - Being able to walk is important | State of health | Tapping on internal self-care repository |
| *“Every day, I am happy and optimistic, I have no worries... I find joy in everything I do. When I read the newspaper daily, watch news, it is enjoyable and I find amusement (in it).”* (Ying Ying, 73)  *“I’m lucky till now, I still got my savings. I am not so bad, not like others… so difficult in life… because now I (am) healthy, because all my children love me, they come and visit me, they still contribute money to me… I am happy.”* (Salinah, 72)  *“I feel that it’s self-discipline…I tried to be in good shape. Every morning, I would take a walk from my house to (shopping centre)…sometimes I will take one big round drop by (supermarket)…reach my favorite point, kopitiam (coffee shop), have coffee and breakfast… although sometimes I hate getting up at that hour, but I self-discipline myself..”* (Keng Gim, 77) | - Having a right positive attitude - Perceptions towards aging - Have open-mindedness - Have optimism - Have self-contentment - Have perseverance to adopt health actions - Have discipline to performing health-promoting acts | Positive disposition |  |
| *“We can follow the exercises which we were taught in school previously and use it to guide us in doing our exercises now.”* (Wah Lee, 75)  *“I listen strictly to doctors… even if your friends were to give you comments, try and weigh whether to follow or not… you must think, take a step back... evaluate whether is it okay to go ahead or not.”* (Peh Chin, 75)  Wah Lee: “*When I was about 60kg, my doctor asked me to lose weight. I told him I swim. He says, “Don’t even drink coffee after your swim. You can gain weigh from drinking it. When you are obese, (it) put pressure to your legs (knees).”*  Eng Hwa: “*Cannot drink coffee?*  Wah Lee: “*Not that you cannot drink. It makes one put on weight.”* (FGD 1) | - Past life experiences influence health behaviours - Knowledge on self-care/chronic disease/food nutrition - Formal and informal sources of health information - Accessibility to health information - Discerning health information | Self-care health knowledge & life experiences |  |
| *“I use the facebook frequently… internet too” (Ching Ai, 75)*  *“I play (handphone) games to improve my memory” (Ah Chye, 79)*  *“Perhaps, there is a particular place whereby people like me…whom they can turn to ask certain questions about the computers, smartphones… not the skills future fund* whereby you attend 3 hours of course... But specific questions… one or two questions… and the best is to be kind of personal, one to one… rather than over the phone…* (Keng Gim, 77) | - Use technological aides to facilitate healthful actions - Keeping up with technological advances | Knowledge of using technological aides |  |
| *“Kinship… although they do not stay with us, they visit us at times and keep in contact. To us, it is a comfort. It affects our physical and mental health. At least we know that our children are still filial to us, they did not abandon us. In terms of mental health, we find solace… feel happy and our moods will be lifted. And we can boast to our friends that our children are filial.”* (Hai Wee, 69)  *“Don’t the daughters think, my mother brought me up, I suckle her milk. She went through those olden days to bring them up and this is the reward given to the mother... years ago our culture is very good, filial piety. But now, it’s lost.”* (Keng Gim, 77)  *“I have been with my wife for 62 years marriage. She is the only one, is my backbone. She encouraged me… not to be distressed, not to worry, take things easy… I treasure spousal love most… Understanding one another throughout these years, until death do us apart. Love one another, understand one another, financially, respect, sickness all these things. There are definitely quarrels between wife and husband... our wife plays a very big part in life”* (Roslan, 78) | - Family is important - Filial piety in children caring for seniors - Children provide instrumental support - Children provide emotional support/bring joy - Spousal relationship is a valued source of support - Quarrels between spouse are inevitable - Being together with spouse brings happiness - Provide caregiving for spouse | Family members | Maintaining and preserving informal social support |
| *“I am living alone now, I am not married. I have a brother but let’s not talk about him… I need friends, it’s very simple. If you call your friend and ask her out, she has a companion, she will be in good spirits. If you are an elderly living alone and you have no friends, (you) watch the sun rises and the sun sets every day (have nothing else to do every day)”* (Nee Mui, 66)  *“I try to mix up (around) more with people. And, I just handed him (another participant) my phone number, since he is also solo (both of them laughed). Make friends, you know. There could be something common between us.” (*Keng Gim, 77)  Di Meng: *“What is glaucoma?”*  Meng Hoe: *“Glaucoma occurs when your optic nerve is damaged.”*  Nee Mui: *“Is it got to do with the eye pressure?”*  Meng Hoe: (in agreement) *“Eye pressure high! Do you know how I was diagnosed? I was taking a nap one day and woke up with... It’s better to do surgery early if have glaucoma.”* (FGD 2) | - Friends are source of companionship - Friends are important when living alone - Friends provide emotional support/ bring happiness - Peers share health information/encourage each other to take health-promoting actions | Friends |  |
| Hai Wee: *“Neighbors are important, because they are the closest to us. If need anything, can help each other out, talk and ask each other out for activities…If you have good relationships with your neighbors, they may look out for you… Now, good neighbors are hard to come by.”*  Chew See: *“Now don’t have such neighbors... it’s more of you mind your own business, I mind my own business.”*  Keng Siew*: “Yah. We usually close our doors when we return home.”* (FGD 5)  *“My neighboring units are rented to Indians. How to communicate with them? Moreover, the (Chinese speaking) younger neighbors will go out to work, seldom at home.”* (Di Meng, 70) | - Neighbours are proximal source of support - Build relationships with neighbours - Not close to neighbours | Neighbours |  |
| *“Money is very important. But it depends how you manage your money… if you don’t look after this during your younger days… it’s very difficult for you… you can’t prepare your old age when you become old already.”* (Roslan, 78)  *“First thing, we must use our money first. Our savings all out first. Then we can get from the government (financial assistance schemes) … because from small, every week hospital hospital (since my son was born, he has been hospitalized weekly). All my savings, all my work, all gone.”* (Hassan, 72) | - Having financial security - Money is a preceding resource - Sources of money | Money | Enabling self by using environmental aides |
| Hai Wee: *“Did you hire someone to help you? Ask your son.”*  Ting Lay: *“Don’t want. My son wants to hire a helper but I say if you hire helper, I will live elsewhere.”*  Interviewer: *“Isn’t this (caring for husband with dementia alone) difficult for you?”*  Ting Lay: “*It is. It does not matter if it is hard for me. I said, wait till I pass away, then you can hire a helper. (laughs) I will continue to care for him as long as I can. Care till I pass on... I don’t like too many people in the house, too complex. I have freedom if I (continue) like this.” (FGD 5)* | - Foreign domestic helper as potential help - Refuse foreign domestic helper | Domestic helper as a potential resource |  |
| *“He has that qualification, he has that experience, surely there is some way he can contribute to the economy. And, for his health also! It’s related. Stress. He misses his wife. Would this be a distraction away from his thoughts from the wife? To (his) limited time? It helps a lot. Furthermore, he still (can) contribute to the economy. He still has economic life! He is not zero.”* (Keng Gim, 77, responding to another participant’s sharing on unsuccessful job interview.)  *“Whenever I go for a job, they will ask blah blah blah.. the last question they ask you, ‘Uncle, how old are you?’… I will tell them, ‘yes, I am 71, 70.’ Then they say, ‘sorry uncle, we don’t accept people with that age.’ ”* (Peh Chin, 75) | - Being able to work is healthy - Reasons for taking up a job - Working as a form of activity participation - Experience of job search | Employment opportunities |  |
| Hai Wee: *“They always encourage seniors to go. Get your friends or neighbors to go together. They encourage us to come down for activities, not to stay at home whole day and watch television, not good for health. But efforts are futile, seems like no one want to go.”*  Lian Lee: *“Some people don’t like to participate in activities. They prefer solitude.”*  Hai Wee: “*…There are massage chairs, newspapers to read, rummy-o (board game)…You do not need to pay for the (massage) equipment. Sometimes, there are talks. On Tuesdays, there is dance class and you don’t have to pay. But people still don’t want to go, even those who live in the same block above the centre.”*  Keng Siew: *“But it’s difficult. Some seniors’ legs are not good, how can they dance?”*  Hai Wee: *“If they cannot dance, they can sing karaoke.”*  Kim Meng: *“I won’t participate in these activities. I don’t like. Different people have different interests. I prefer to walk around on my own and spend time alone… they are afraid that some seniors keep staying at home…”*  Ting Lay: *“It’s better to participate… if don’t participate, can get dementia easily. Some people don’t participate and just stay at home… my goodness, it’s better to go. But if you don’t have time, it can’t be helped. I will go if I have the time.”* (FGD 5)  *“Seniors living alone need someone to visit them weekly, else will grow worms (decompose-become useless and expire soon). So now we do house visits for them and it is a form of activity for us. ‘Uncle, have you eaten? Have you taken your medicine?’ That’s what we do… this is important, to have volunteers to visit… Now we still have the energy... maybe another two more years, I also need someone to visit me...”* (Nee Mui, 66)  *“I am on meals-on-wheels, by XXX (non-profit organisation)… When I need to see a doctor, there is a van arranged to transport me there. I have a (mobility) scooter too (to move around). They have my medical appointment records, I don’t need to tell them. When the date is up, they will call to tell me… There are volunteers and 3 social workers taking care of me.”* (Ah Leong, 78) | - Community-based centres/organisations as resources for activity participation - Barriers to community activity participation - Volunteering at community-based centres/organisations - Receiving help from volunteers - Receiving help from social services | Community service organisations and volunteerism |  |
| *“At the moment, the ministry is doing a lot of things, helping the senior citizens, the people a lot. I should say I appreciate that very much... so their (the government) vision, we follow.”* (Roslan, 78)  *“Now if we want to keep our minds active, we can use the $500 (SkillsFuture) credit given by the government… you just need to register for the class you like… I have used the $500… to attend courses on how to do business, how to engage others in conversations, and English classes too.”* (Ching Ai, 75) | - Appreciate government’s efforts in providing services/schemes to elderly - Place faith and dependence on government for service provision - Government support schemes as behaviour motivator | Government support schemes |  |
| *“There is a private clinic at the residential block, you can test your blood there... if you don’t want to test it at government (poly)clinics, you can go to private doctor.”* (Ting Lay, 75)  *“If anything happens, just go and see the doctor. Even if it’s nothing serious, I will see. When the doctor said my leg has recovered and I can be discharged, then my leg is okay.”* (Ah Leong, 78)  *“The doctor says I have diabetes based on the blood results.. you just based on the machine to test, how you know? There are no ants in my urine.. I have heard from many people, from talks on that. If you sustain a cut, the wound will rot and not heal. I accidentally cut my finger while cutting my fingernails, there is only blood that ooze from it. I am still good from it… how to prove that I have diabetes?”* (Lian Lee, 77) | - Have access to affordable healthcare - Trust/rely health care professions for treatment/advice | Healthcare services |  |
| *“Our living environment also need more exercise facilities which are suitable for seniors. It will be a concern if lack of such (facility)... to do exercise to keep fit, such as those physical therapy equipment.”* (Meng Hoe, 75)  “… environment must be good. There is some elderly who live alone and they keep many items till their house are cluttered… that is unhygienic… dirty and harmful to body” (Wah Lee, 75)  *“Now the government helps those seniors living alone, can apply for various schemes to do up anti-slip tiles in the toilets and bathrooms. And the grab bars too.”* (Hai Wee, 69) | - Having senior-friendly amenities and public spaces - Have safe home living environment | Senior-friendly physical amenities |  |
| *“I ordered my coffee and left my last capsule of high blood pressure and high cholesterol packet medicine on the table… and here come.. I thought she is a table cleaner. And she said, ‘Uncle, you are taking certain medicine. Can I offer you some water?’ That touched me… all these years I have visited kopitiam (coffee shop), I have never encountered such a thoughtful pleasant lady… Now, look at my age, never have I experienced such kind of encounter. Wouldn’t it be nice? You know, and I felt very pleased. And when a person feels pleased, it is also good for the health. Isn’t it? So this should be spread.”* (Keng Gim, 77)  When I go in the bus, and they see me with heavy trolley on me, they still help me. Most of them they’re young, younger generation lah. Then when I went to the MRT, also, they say, they give me a seat. Because I know I can’t, I walk like a penguin.. (laughs) (Salinah, 72) | - Having people around to be ‘elderly-friendly’ - Experiences of others recognising their needs as seniors | Civic-mindedness towards seniors |  |
